# Autologous Bone Versus Xenograft and Their Combination in Vertical Ridge Augmentation: An Analysis of Graft Resorption and Implant Survival—A Systematic Review

**DOI:** 10.3390/dj14060321

**Published:** 2026-05-25

**Authors:** Ana Rosa Otero-Cruz, María Pilar Pecci-Lloret, Nuria Pérez-Guzmán, Ali El-Yahyaoui El-Akrout, Juan Antonio Ruiz-Roca

**Affiliations:** Department of Dermatology, Stomatology, Radiology and Physical Medicine, Hospital Morales Meseguer, Medicine School, University of Murcia, 30100 Murcia, Spain; anarosa.oteroc@um.es (A.R.O.-C.); mariapilar.pecci@um.es (M.P.P.-L.); ali.ely@um.es (A.E.-Y.E.-A.); jaruizroca@um.es (J.A.R.-R.)

**Keywords:** vertical bone augmentation, autologous bone, xenograft, bone resorption, implant survival

## Abstract

**Background**: The integrity of the alveolar ridge is compromised by tooth loss or trauma, initiating chronic resorption that alters bone morphology. Vertical bone augmentation (VBA) is a complex procedure required to rehabilitate severe atrophy. Currently, there is debate regarding the effectiveness of autologous bone versus xenografts and their combination to optimize bone regeneration and ensure dimensional stability. **Objectives**: To synthesize evidence on the efficacy of VBA by comparing autologous bone, xenografts, and their combination. Specifically, to evaluate vertical bone gain, volumetric stability and resorption, implant survival, complications, and whether combined grafts offer clinical advantages. **Methods**: A systematic review was conducted following PRISMA 2020 guidelines. MEDLINE, Scopus, SciELO, and Web of Science were searched up to January 2026. Risk of bias was assessed, using RoB 2 for randomized studies and ROBINS-I for non-randomized studies. **Results**: From 1517 initial records, 9 studies were included and 3 showed high risk of bias. Iliac crest grafts achieved the greatest vertical bone gain but also exhibited higher resorption compared to calvarial grafts. Xenografts (Bio-Oss) demonstrated superior volumetric stability, maintaining 10–13% more residual volume than autologous blocks. The most frequent complication was soft tissue dehiscence. **Conclusions**: VBA is an effective procedure. The combination of autologous bone and xenograft may represent a balanced approach, providing both biological potential and volumetric stability. Graft origin significantly influences outcomes and morbidity. However, current evidence is limited by methodological heterogeneity and small sample sizes, highlighting the need for well-designed clinical trials with standardized protocols and long-term follow-up.

## 1. Introduction

The integrity of the alveolar ridge may be compromised by multiple factors, such as bone remodeling following tooth extractions, periodontal disease, trauma, or the resection of tumor lesions. These events initiate a process of chronic resorption that alters the morphology of the ridge in all its dimensions, which is especially notable during the first 3 to 6 months after tooth loss [1,2].

Bone tissue is an adaptive structure whose mechanical integrity is regulated by the constant interaction between functional load and tissue deformation. Under conditions of disuse or loss of biomechanical stimulus, the biological feedback system detects that mechanical stresses fall below the minimum maintenance threshold. In this scenario, physiological balance is disrupted, leading to a negative bone balance in which the rate of osteoclastic resorption exceeds the osteoblasts’ capacity for synthesis [3].

Given the inherent characteristics of vertical defects, augmentation procedures require specific planning and technical execution that must be clearly defined. The biological success of regenerative materials depends not only on their intrinsic properties, but also on the surgical methodology employed according to the severity of the atrophy [2,4].

The vascular compromise present in vertical defects is one of the main determinants in soft tissue management. To ensure success, it is imperative to avoid excessive pressure that could compromise graft stability and its revascularization [5]. Surgical management should prioritize tension-free primary closure through periosteal releasing incisions, as excessive flap tension is the main cause of dehiscence and membrane exposure.

A distinction can be made between Grade I vertical defects, or mild vertical loss (≤3 mm), in which the expected outcome is predictable and more conservative alternatives may be used, such as orthodontic tooth movement or the placement of short implants [4]. In Grade II (moderate) vertical defects (4–6 mm) and Grade III (severe) defects (>7 mm) [4], it is often necessary to resort to more complex surgical techniques, such as some of those mentioned below:

- Osteotomy technique (“sandwich technique”—inlay or interpositional): This consists of performing a horizontal osteotomy of the residual alveolar ridge and placing osteosynthesis material between the two fragments in a sandwich configuration, or stabilizing the fragments with the implant itself [6,7].
- Onlay bone grafts: For small grafts, donor bone from intraoral sites such as the mandibular ramus, the chin, or edentulous areas can be used. To obtain larger bone volumes, donor sites such as cancellous bone from the iliac crest and calvarial bone are utilized [8]. However, this technique presents several limitations, such as a high resorption rate (particularly with extraoral bone) and postoperative morbidity associated with the donor site [1,9].
- Tenting technique (or tent-pole): Screws are used to support bone blocks or soft tissue fixed over the alveolar ridge in order to create and maintain a stable space, which is subsequently filled with particulate graft, preventing the collapse of the soft tissues. [10]. The so-called Barbell Technique uses a device with a “tent-pole” effect, initially designed for horizontal buccolingual augmentation, but it has also been applied in vertical augmentations in combination with particulate grafts and reinforced membranes, blending onlay and inlay techniques [11].
- Sinus elevation: In posterior maxillary cases with severe vertical crestal bone atrophy and enlarged maxillary sinus cavities, a sinus approach is performed—either through a crestal (horizontal) technique or a lateral window—where the Schneiderian membrane is elevated, maintaining the created space by means of graft material placement [12,13].
- Guided bone regeneration (GBR): One of the most widely used techniques for vertical bone augmentation. The protocol is based on the principle of “cell exclusion” [14]. It consists of filling the defect with particulate bone, which is then covered by a membrane that acts as a barrier to isolate the graft from epithelial and connective tissue, allowing osteoblasts to regenerate bone separately from other cell types [1,15].

Despite the widespread clinical use of different materials for vertical bone augmentation, the available literature shows considerable methodological heterogeneity and a lack of direct comparative studies that simultaneously evaluate the use of autologous bone, xenografts, or a combination of both. For this reason, clinical decision-making based on objective criteria is challenging, especially in cases of severe alveolar atrophy.

Therefore, the general objective of this systematic review is to synthesize the available scientific evidence on the clinical efficacy of vertical bone augmentation in patients with severe alveolar atrophy of the maxilla and/or mandible, directly and indirectly comparing the outcomes obtained with autologous bone grafts, xenografts, and their combination, within the context of the different surgical techniques employed.

## 2. Materials and Methods

The systematic review was conducted following the PRISMA 2020 protocol (Preferred Reporting Items for Systematic Reviews and Meta-Analysis) [16] for systematic reviews (Supplementary File S1). It was registered in PROSPERO under registration number CRD420251207069 on 8 November 2025.

As a framework for the systematic review, the following PICO question was established:

P: Patients with severe vertical bone atrophy of the maxilla or mandible, requiring rehabilitation with implants.

I: Vertical bone augmentation procedures performed using 100% autologous bone, 100% xenograft, or a combination of autologous bone + xenograft.

C: Direct comparison between materials when available/indirect comparison between groups according to the type of graft used.

O: Net vertical bone gain (in mm), vertical graft resorption rate (before and after loading), implant success/survival, and postoperative complications [mesh exposure, infection, graft loss, hospital stay]).

This resulted in the following research question:

What clinical outcomes are achieved in vertical bone gain when using autologous bone, xenograft, or their combination in patients with severe vertical bone atrophy of the maxilla or mandible?

### 2.1. Eligibility Criteria

Articles were included according to the following criteria: (I) human patients aged over 18 years; (II) requiring vertical bone augmentation; (III) use of autologous bone, xenograft, or a combination of both in different regenerative techniques; (IV) comparative clinical studies (randomized or non-randomized) including at least two intervention groups or a control group; (V) clinical and radiographic follow-up of at least 4 to 6 months after the bone augmentation phase, covering at least up to the evaluation of net bone gain, implant placement, or the initial phases of prosthetic rehabilitation; and (VI) studies in English or Spanish.

The exclusion criteria were as follows: (I) studies requiring exclusively horizontal augmentation; (II) use of allografts or synthetic biomaterials; (III) articles consisting of single case reports or case series; and (IV) literature reviews, systematic reviews, letters, or opinion articles.

### 2.2. Search Strategy

#### 2.2.1. Information Sources

An initial literature search was conducted on 6 November 2025, and was updated on 12 January 2026 in the databases MEDLINE (using the PubMed search engine), Scopus, SciELO, and Web of Science.

#### 2.2.2. Search Terms

The search strategy combined controlled vocabulary (MeSH terms, used in MEDLINE via PubMed) and free-text keywords adapted for each database (Scopus, SciELO, and Web of Science). The search terms included the following: “vertical ridge augmentation”, “vertical bone augmentation”, “bone grafting”, “bone regeneration”, “autologous bone”, “autologous bone”, “xenograft”, “bovine bone”, “Bio-Oss”, “resorption”, “bone loss”, “dimensional stability”, “volume maintenance”, “maxilla”, and “mandible”. The full search strategy was as follows: ((“vertical ridge augmentation” OR “vertical bone augmentation” OR “bone grafting” OR “bone regeneration”) AND (“autologous bone” OR “autologous bone” OR “xenograft” OR “bovine bone” OR “Bio-Oss”) AND (“resorption” OR “bone loss” OR “dimensional stability” OR “volume maintenance”) AND (“maxilla” OR “mandible”)).

### 2.3. Study Selection and Data Extraction

Study selection was performed by two independent reviewers (AROC and MPPL) who screened titles and abstracts according to the predefined eligibility criteria. Any disagreements were resolved through a third reviewer (NPG). Data extraction was conducted independently by two reviewers using a standardized data collection form specially designed for this review. The extracted variables included: study design, sample size, anatomical location, surgical technique, type of graft material, fixation method, vertical bone gain (mm), graft resorption (mm and/or %), implant survival and success rate, follow up duration and reported complications.

### 2.4. Risk of Bias Assessment

Randomized clinical trials were evaluated using the RoB 2 tool, while non-randomized studies were assessed using the ROBINS-I v2 tool. The assessment was performed according to the guidance provided for each tool. For ROBINS-I, studies were evaluated across the predefined domains, and the overall risk of bias was determined based on the highest level of bias identified in any critical domain. For RoB 2, domain-level judgments were combined to reach an overall risk of bias following the recommended algorithm. It was performed by two reviewers (AROC and MPPL) and any discrepancies were resolved through discussion and consensus; when an agreement could not be reached, a third reviewer was consulted (NPG).

## 3. Results

### 3.1. Study Selection and Flow Diagram

**Figure 1:**
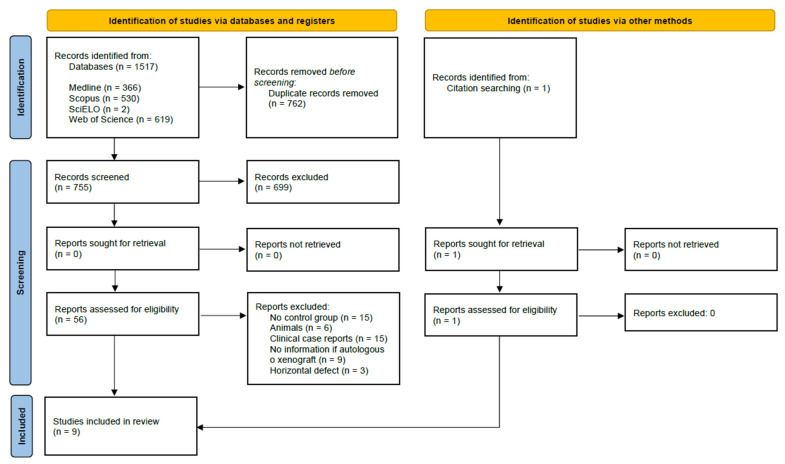
Flow diagram PRISMA 2020.

The search yielded a total of 1517 preliminary references related to vertical bone height gain required in implantology: 366 from MEDLINE, 530 from Scopus, 2 from SciELO, and 619 from Web of Science. After automatically removing 453 duplicates and 309 manually, the remaining 755 articles were screened. One additional article was manually identified from the references of another study. Of these, 699 were excluded after reviewing the title and abstract. The remaining 57 articles were assessed in full text. Among them, 15 were excluded for lacking a control group for comparison, 6 for being conducted in animal teeth, 15 for being case series or case reports, 9 for not using either autologous or xenograft material, and 3 for addressing horizontal regeneration. Finally, 9 articles were selected for qualitative analysis (Figure 1).

### 3.2. Risk of Bias Assessment Results

To evaluate the methodological quality of non-randomized studies, the ROBINS-I tool (Risk of Bias in Non-randomized Studies of Interventions) was used, and the results are detailed in Table 1. Six of the included articles [6,8,13,17,18,19] were assessed using this scale. Of the evaluated studies, three were classified as having an overall moderate risk of bias [8,17,18]. In contrast, the remaining three studies [6,13,19] were rated as having a serious overall risk of bias. All studies were retained in the qualitative synthesis; however, those with a serious risk of bias were interpreted with caution due to concerns in key domains that may compromise internal validity.

Finally, three studies included in this review were randomized in vivo clinical trials. Table 2 presents the results of the risk of bias analysis for each domain. According to the criteria of the RoB 2 tool, all three articles [11,20,21] showed some concerns (moderate risk) in their overall assessment. As no critical risks of bias were identified, all were included in the results and discussion of this study.

Due to the heterogeneity of the included studies in terms of design, surgical techniques, graft materials, and outcome reporting, a quantitative synthesis (meta-analysis) was not feasible. Therefore, a qualitative synthesis was performed, and comparisons between interventions were based on a structured narrative analysis of the reported outcomes.

### 3.3. Population Characteristics, Surgical Sites, and Anatomical Location

The studies included in this review comprised a total population of 120 patients, in whom 146 surgical sites were treated using various vertical bone augmentation techniques. Sample sizes ranged from pilot studies with 6 patients to investigations including up to 40 subjects (Supplementary File S2).

Regarding the anatomical location of the defects, a balanced distribution between the maxilla and mandible was observed:

- Posterior mandible exclusively: 54 treated sites [18,19,21].
- Posterior maxilla exclusively: 79 treated sites [6,11].
- Mixed location (both jaws): 126 sites distributed (Supplementary File S2) [8,13,17,20].

### 3.4. Surgical Techniques and Graft Materials

In Supplementary File S2, the interventions are grouped into four main approaches:

- Onlay technique (appositional): The graft material is placed directly onto the bone crest to achieve vertical height gain [6,8,18,19,20].
- Inlay (interpositional) technique [21].
- Fixation devices and membranes: Predominantly resorbable collagen membranes or cortical bone barriers were used to protect the graft [11,13,20,21].
- Specialized techniques: Protocols such as cortical tenting (where the block acts as a barrier) and the Barbell Technique were identified, using titanium and PEEK devices to stabilize autologous micrografts obtained by mechanical disaggregation [11,18].

### 3.5. Vertical Bone Gain Achieved (mm)

Increases in alveolar ridge height varied significantly depending on the technique and graft origin (Supplementary File S2). Iliac crest grafts provided the highest mean gains, with reported values of 13.35 mm [8] and 3.1 mm [20]. In comparison, calvarial bone achieved a significantly lower gain of 4.22 mm [8].

In the posterior mandible, the layered onlay technique achieved a mean gain of 4.48 mm, while cortical tenting reached 5.2 mm. Block onlay approaches in the mandible achieved up to 6.23 mm [18].

When stratified by anatomical location, in combined procedures in the posterior maxilla, the Barbell Technique achieved an inlay gain of 8.8 mm and an onlay gain of 1.5–1.8 mm [8]. Other interpositional studies confirmed that these techniques provide sufficient height to place implants of 9–10 mm in length [21]. Gultekin et al. (2017) [6] reported greater vertical gain with autologous iliac crest block grafts (8.31 mm) compared to guided bone regeneration (GBR) using a mixture of particulate autologous bone and xenograft (5.07 mm); however, these findings should be interpreted with caution, as the study was assessed as having a serious risk of bias.

### 3.6. Graft Resorption Rate (%) and Vertical Resorption (mm)

Dimensional stability during healing was strongly influenced by the graft material (Supplementary File S2). Autologous iliac crest blocks showed the highest resorption [6,8], which worsened significantly (severe resorption) when implant placement was delayed beyond 9 months. In contrast, calvarial bone demonstrated superior stability.

Resorption patterns also varied depending on the technique. In the mandible, inlay xenografts showed 10–13% more residual volume than autologous blocks [21]. Cortical tenting reduced vertical loss to 18.4% (1.17 mm), compared to 28.1% (1.75 mm) with conventional onlay [18].

### 3.7. Number of Implants and Implant Success/Survival

A total of 737 dental implants were placed across the analyzed studies. The sample distribution was heterogeneous, ranging from studies with 174 implants [6], to pilot studies with 12 units [18]. Overall survival rates were very high, generally ranging between 97.3% and 100% in most studies (Supplementary File S3).

The results according to the graft material revealed notable differences:

- Autologous bone: Demonstrated consistently high survival rates, with values of 98.7% [20] and 100% in various iliac graft protocols [8].
- Xenografts: Showed a critical reduction in survival to 82.8% when equine blocks were used in the mandible due to integration failures [20]. In contrast, bovine blocks (Bio-Oss) used in inlay techniques showed greater predictability, achieving 97.3% survival after one year of functional loading [21].
- Specialized techniques: the Barbell Technique reported a survival rate of 98% [11], with only one early failure among 50 implants.

### 3.8. Follow-Up Time and Marginal Bone Loss (MBL)

Supplementary File S3 shows high variability in follow-up periods after prosthetic rehabilitation, ranging from preliminary studies of 4 months [18] to long-term follow-ups with a mean of 6.2 years [17] (range 3–12 years).

Regarding marginal bone loss after functional loading (Supplementary File S3):

- Mean values: During the first year, bone remodeling ranged between 0.59 mm and 1.3 mm.
- Influence of technique: Direct comparisons showed losses of 0.82 mm for autologous bone versus 0.59 mm for Bio-Oss, with no statistically significant differences [21].
- Critical long-term cases: In follow-ups exceeding 10 years, mean MBL reached 2.18 mm, with maximum resorption up to 3 mm localized exclusively in molar regions [17].

### 3.9. Complications, Morbidity, and Graft Failure Rate

Recipient site complications (dehiscence, infection, fractures):

The most frequent complication reported (Supplementary File S4) was soft tissue dehiscence with subsequent exposure of the graft or membrane, with a notably high incidence of 13 cases exclusively in the block xenograft group (XB) [20], whereas the autologous group showed only minor complications. Five cases of dehiscence led to partial graft failure in two of them [8].

Severe complications such as fractures were mainly associated with autologous block techniques or interpositional procedures. One case of total graft failure was reported due to fracture of the osteotomized bone segment during fixation, leading to infection [21]. In contrast, other studies reported no inflammatory or infectious complications during follow-up [17,18].

Donor site complications (pain and paresthesia):

Donor site morbidity was significantly higher in cases requiring extraoral bone. Patients receiving iliac crest grafts experienced more persistent moderate pain [20], with 10 patients still reporting pain 10 days postoperatively, including one case of limping that persisted for 6 months. The iliac site was also described as technically demanding and more painful compared to bone substitutes (Supplementary File S4) [21].

### 3.10. Hospital Stay and Total Graft Failure Rate

The need for hospitalization was directly linked to graft harvesting (Supplementary File S4). Patients undergoing iliac crest grafting required hospital stays ranging from 3.1 nights [20] to 5 days [8]: the latter was for intravenous antibiotic administration. One study reported hospitalization of all patients for 3 days [21], whereas less invasive techniques such as micrografts were performed on an outpatient basis.

Finally, total graft failure rates showed critical variation:

- Block xenografts (XB): Highest failure rate, reaching 50% overall [20], with 100% failure in the mandible due to lack of integration.
- Autologous bone: Showed occasional failures related to technical complications, with rates around 10% [21].
- Specialized techniques: Studies [11,17,18] reported a 0% graft failure rate, establishing them as highly predictable procedures for implant rehabilitation.

## 4. Discussion

The included studies exhibited substantial clinical and methodological heterogeneity, including differences in surgical techniques (onlay, inlay, tenting, GBR), graft materials (autologous bone, xenografts, and combinations), anatomical locations (maxilla vs. mandible), and follow-up durations. These variations introduce important confounding factors that limit direct comparison between studies and may influence the reported outcomes.

### 4.1. General Considerations on Vertical Bone Augmentation (VBA)

Vertical bone augmentation (VBA) is established in the contemporary literature as one of the most demanding surgical procedures in implant rehabilitation. Its complexity lies primarily in the need to create and maintain a stable space against constant soft tissue pressure, within a biologically unfavorable environment for revascularization.

The results analyzed in this systematic review allow for a critical evaluation of the effectiveness of different graft materials, surgical techniques, and their impact on both implant survival and patient morbidity.

### 4.2. The Resorption Paradox: Autologous Bone vs. Xenograft

Autologous bone is widely considered the gold standard due to its osteogenic and osteoinductive properties. In the present review, the analyzed results reveal a marked susceptibility to volumetric resorption. While calvarial grafts (intramembranous origin) showed notable stability with only 8.44% loss, iliac crest grafts (endochondral origin) reached resorption rates of 24.16%. This difference, corroborated by Mertens et al., suggests that graft microarchitecture and density are more decisive than embryological origin in resisting soft tissue pressure during healing [8].

In the present review, xenografts demonstrated greater volumetric stability. This observation is supported by previous studies [22], which described xenografts (specially DBBM or Bio-Oss) as an inert filler that remains within the bone tissue and is only utilized or resorbed during extensive remodeling. They observed that unlike cortical autologous bone (which lost up to 50% of its length due to peripheral resorption), xenograft blocks maintained their original dimensions during healing, albeit with moderate internal new bone formation [22].

Despite their low resorption rate, xenografts are not static materials. Simion et al. histologically observed resorption lacunae and osteoclasts adjacent to Bio-Oss particles, indicating participation in a physiological remodeling process with alternating phases of demineralization and remineralization [6,23]. This process of “progressive substitution” allows intimate integration with newly formed lamellar bone. Interpositional studies confirm that Bio-Oss blocks maintain 10–13% more residual volume than autologous blocks [2].

Based on the findings of the present review, the combination of autologous bone and particulate xenograft may represent a balanced approach. Previous studies have also suggested the use of a 1:1 mixture of autologous bone and particulate xenograft is recommended in GBR techniques, combining biological induction with mechanical resistance against soft tissue pressure [23]. According to Gultekin et al., this combination reduced resorption to 15.87% (compared to 41.62% in autologous block grafts) and appeared to eliminate the influence of individual factors such as age or sex on volumetric outcomes [6].

Biologically, this combination fulfills the requirements of the triad: the autograft provides cells and inductive capacity, while DBBM ensures scaffold stability and long-term volume maintenance (4). Long-term follow-ups of up to 10 years, such as those by Khoury and Hanser, demonstrate that regenerated bone maintains 91.7% stability, comparable to native bone [24].

The management of peri-implant gaps (so-called “jumping gaps”) provides additional insight into the biological behavior of graft materials. A recent systematic review by Ahamed SK et al. (2025) [25] highlighted the importance of space maintenance and the use of slowly resorbing biomaterials, such as xenografts, to preserve peri-implant bone volume. These findings support the results of the present review, where xenograft-containing approaches demonstrated improved volumetric stability and reduced resorption rates.

### 4.3. Influence of Surgical Technique: Inlay vs. Onlay

The morphology of the bone defect plays a decisive role in selecting the appropriate surgical technique for predictable vertical augmentation [26]. In the included studies, interpositional (inlay) techniques, such as the sandwich osteotomy, stand out for ensuring rapid graft incorporation due to excellent vascular supply from adjacent vital bone segments connected to their periosteum [27]. Felice et al. reported this approach as highly effective for rehabilitating the atrophic mandible with low final resorption rates. This is consistent with the previous literature; AbuHarb et al. confirmed that the inlay design is a viable and predictable technique, achieving stable gains of approximately 4.46 mm with minimal postoperative complications [27].

In contrast, onlay block grafts face greater biological challenges, as flap pressure and increased distance from vascular supply can lead to resorption rates of up to 28.1% [18]. Sass et al. noted that the risk of bone loss is significantly higher in augmentations toward the oral cavity (onlay) compared to protected environments such as sinus elevation (inlay) [17].

In direct comparisons, Barone et al. observed that although onlay blocks achieve greater initial height (7.4 mm), inlay techniques (6.0 mm) show similar volumetric remodeling and higher success (93.8% vs. 82.4%), suggesting that interpositional techniques are less aggressive but more technique-sensitive [19].

### 4.4. Biological Limits: Analysis of Graft Failure

The predictability of vertical augmentation depends not only on the material but also on the vascular potential of the recipient bed. The drop in implant survival to 82.8% reported by Pistilli et al. highlights the biological limits of block xenografts. The 100% failure rate in the posterior mandible is explained by its relative hypovascularization compared to the maxilla.

Since equine grafts lack vital cellular components, their integration depends entirely on peripheral angiogenesis, which the mandibular bed could not provide. Consequently, the graft behaves as an inert body, leading to asymptomatic dehiscence and complete graft loss. Conversely, Felice et al. demonstrated that the same material (Bio-Oss) can be successful in the posterior mandible when used as an inlay graft [2].

Pistilli et al. also highlighted the risk of using pre-shaped xenograft blocks on 3D models, where 5 out of 6 failed, possibly due to contamination prior to surgery [20]. Additionally, Felice et al. warned that fractured Bio-Oss blocks should be discarded, as fragmentation can lead to collapse of the elevated segment [21].

### 4.5. Mechanical Stability for Treatment Success

The literature confirms that absolute immobilization is essential to protect angiogenesis. Morad et al. demonstrated that cortical tenting reduced resorption from 28.1% to 18.4% by acting as a mechanical scaffold against soft tissue pressure. This biomechanical approach has been optimized with wide-head screws and the Barbell Technique, which stabilizes micrografts and xenografts in high-pressure environments, achieving up to 57.5% vital bone formation without complex meshes [18]. Kim et al. reported vital bone formation between 21.2% and 57.5% when using a single screw to resist soft tissue contraction, eliminating the need for additional fixation systems [28].

### 4.6. Donor Site Morbidity and Patient Burden

The studies confirm that graft material selection is the main determinant of postoperative morbidity. Iliac crest grafts, although effective for large reconstructions, involve significant surgical burden [8]. Pistilli et al. reported an average hospital stay of 3.1 nights, compared to outpatient procedures for bone substitutes. These grafts are also associated with persistent pain and gait disturbances [20]. Felice et al. emphasized that extraoral autografts are more costly, require general anesthesia, and are less accepted by patients compared to Bio-Oss blocks [2].

In contrast, intraoral grafts (ramus or symphysis) show significantly lower morbidity. Khoury and Hanser reported that retromolar grafting avoids hospitalization and results in mild to moderate pain with rapid recovery and no long-term functional impairment [29].

Paresthesia and neurosensory alterations are common but mostly transient. Barone et al. reported temporary paresthesia resolving within 1–2 months [19]. Gultekin et al. documented a 5% incidence in iliac crest donor sites [6], and Novy et al. reported mental nerve dysfunction resolving within 2–3 months [30]. The risk in intraoral donor sites is also around 5%, highlighting the importance of minimally invasive techniques and piezosurgery to protect neurovascular structures [24].

### 4.7. Implant Survival and Marginal Stability After Loading

The analyzed results show excellent implant survival rates, consistently between 92% and 100%. Long-term studies up to 10 years, such as Khoury and Hanser, reported 98.1% survival in regenerated bone, demonstrating functional equivalence to native bone [24].

Regarding MBL, most authors agree that the greatest remodeling occurs during the first year of loading and stabilizes thereafter. Cucchi et al. reported 0.67 mm (PTFE) and 0.61 mm (titanium mesh) after one year [14], while Urban et al. observed 1.01 mm at 12 months, remaining stable over 6 years [4].

### 4.8. Limitations and Future Perspectives

Despite advances in vertical bone augmentation techniques, the literature presents significant methodological limitations. Most studies involve small sample sizes and single-center designs, limiting statistical power and generalizability. Additionally, many studies are retrospective, hindering standardized evaluation of long-term volumetric remodeling and biological stability.

High heterogeneity in surgical protocols, graft materials, and implant systems introduces confounding variables, complicating direct comparisons. There is also a lack of long-term (>10 years) follow-up data for many modern techniques. Given the heterogeneity of the included studies and the qualitative nature of this review, these findings should be considered exploratory and hypothesis-generating, rather than definitive.

Furthermore, patient-reported outcome measures (PROMs) are often not systematically assessed, limiting our understanding of pain and quality of life.

Future perspectives in bone regeneration are increasingly focused on minimally invasive and biologically driven approaches. Among these, tunnel techniques have emerged as a promising alternative, as they preserve flap vascularization by minimizing periosteal disruption and avoiding releasing incisions, potentially reducing the risk of wound dehiscence and improving regenerative outcomes. Recent evidence, such as the case series by G. D’Albis et al. (2026) [31], has reported favorable results in ridge augmentation using xenogeneic bone, hyaluronic acid, and dermal matrix through a tunnel approach.

## 5. Conclusions

The analyzed literature allows for a comparison of the effectiveness and complications of vertical bone augmentation using autologous bone, xenografts, or their combination. Within the limitations on this systematic review, the following conclusions can be drawn.

Vertical bone augmentation is a predictable procedure; however, there is no single ideal material for all clinical scenarios. Individualized selection is required based on defect severity and patient tolerance to morbidity. The combination of autologous bone and xenograft may represent a promising and balanced approach, potentially combining biological properties with improved volumetric stability.

Current evidence is limited by high methodological heterogeneity and lack of long-term studies, as well as insufficient evaluation of patient-reported outcomes. Further randomized clinical trials and standardized protocols are necessary to establish definitive clinical recommendations. These findings should be interpreted with caution and considered hypothesis-generating, rather than definitive.

## Figures and Tables

**Table 1:** Risk of bias in non-randomized studies of interventions.

Articles	D1	D2	D3	D4	D5	D6	Overall
**Gultekin and cols., 2017** [6]	S	L	S	M	L	M	S
**Mertens and cols., 2013** [8]	M	L	M	M	L	M	M
**Sass and cols., 2022** [17]	M	L	M	M	M	M	M
**Urban and cols., 2009** [13]	M	L	S	M	M	M	S
**Morad and cols., 2013** [18]	L	L	M	L	M	M	M
**Barone and cols., 2017** [19]	S	L	M	M	L	M	S

S: Serious. L: Low. M. Moderate. D: Domain.

**Table 2:** Risk of bias in randomized studies of interventions.

Articles	D1	D2	D3	D4	D5	Overall
**Cosmo and cols., 2024** [11]	L	L	L	L	M	M
**Pistilli and cols., 2014** [20]	L	L	L	L	M	M
**Felice and cols., 2009** [21]	L	L	L	L	M	M

S: Serious. L: Low. M. Moderate. D: Domain.

## Data Availability

No new data were created or analyzed in this study.

## Supplementary Materials

The following supporting information can be downloaded at: https://www.mdpi.com/article/10.3390/dj14060321/s1, File S1: PRISMA 2020 checklist. File S2: Studies characteristics. File S3: Implants information. File S4: Implants complications.
