# Supplementary material for: Autologous Bone Versus Xenograft and Their Combination in Vertical Ridge Augmentation: An Analysis of Graft Resorption and Implant Survival—A Systematic Review
Source: Dent J (Basel). 2026 May 25;14(6):321. doi: 10.3390/dj14060321 (PMC13297769; doi:10.3390/dj14060321)
Supplement: Supplementary file 1 [file dentistry-14-00321-s001.zip › File S2 Studies characteristics.pdf]

Author (Year)	Study Design	Sample (patients / sites)	Anatomical Location	Surgical Technique	Graft Material	Membrane / Device	Vertical Bone Gain (Mean)	Resorption Rate (%) / Vertical Resorption (mm)
**Pistilli et al. (2014)**	Randomized controlled clinical trial	40 patients (20 per group) / 40 sites	Maxilla (n=28) and mandible (n=12)	Onlay bone blocks	Autologous (AB) (mandibular ramus or iliac crest) vs. Xenograft (XB – equine)	Titanium screws (1.2/1.5 mm), osteosynthesis plates, and resorbable equine cortical bone barriers with tacks	Autologous: 3.1 ± 2.9 mm; Xenograft: 3.3 ± 3.7 mm	Severe resorption observed in iliac crest grafts when implant placement was delayed >9 months
**Mazuchelli et al. (2024)**	Prospective randomized controlled clinical trial	19 patients / 24 sites	Posterior maxilla	Barbell Technique (Inlay + Onlay)	Control group: Inlay: Bio-Oss; Onlay: Bio-Oss + autologous. Test group: Inlay + Onlay: Bio-Oss + autologous micrograft	Barbell device (Titanium/PEEK), 14 mm screws, collagen membranes	Control group: Inlay: 8.95 ± 1.79 mm; Onlay: 1.72 ± 1.56 mm. Test group: Inlay: 8.65 ± 2.62 mm; Onlay: 1.52 ± 1.50 mm	Similar tomographic outcomes across all groups at 6 months
**Felice et al. (2009/2010)**	Randomized controlled clinical trial	10 patients (split-mouth) / 20 sites	Posterior mandible	Interpositional block grafts (Inlay)	Autologous (iliac crest) vs. Bio-Oss (inorganic bovine block)	Titanium miniplates and miniscrews, covered with resorbable collagen membrane	Sufficient gain for placement of 9–10 mm implants	Xenograft (Bio-Oss): 10–13% more residual graft than autologous group

Author (Year)	Study Design	Sample (patients / sites)	Anatomical Location	Surgical Technique	Graft Material	Membrane / Device	Vertical Bone Gain (Mean)	Resorption Rate (%) / Vertical Resorption (mm)
**Morad (2013)**	Prospective pilot study (non-randomized)	6 patients / 12 sites	Posterior mandible	1. Cortical tenting vs. 2. Layered onlay technique	1. Autologous + xenograft (50:50 particulate) vs. 2. Autologous + xenograft coverage	Fixation screws (10–12 mm) and microscrews (cortical block acts as membrane)	1. Tenting: 5.2 mm (final, 4 months). 2. Onlay: 4.48 mm (final, 4 months)	1. Tenting: 18.4% (1.17 mm). 2. Onlay: 28.1% (1.75 mm)
**Sass et al. (2022)**	Retrospective observational comparative study (non-randomized)	22 patients / 23 sites	Maxilla (n=20), mandible (n=3)	• Sinus elevation (INLAY) • Vertical augmentation toward oral cavity (ONLAY)	Autologous monocortical bone blocks (chin, mandibular ramus, calvaria, or iliac crest)	—	Not directly quantified; sufficient gain for implant placement in all cases	Mean marginal bone loss: 2.18 mm (± 0.75) over 3–11 years. Ridge augmentation: 76% showed resorption. Sinus elevation: 8%
**Mertens et al. (2013)**	Retrospective observational comparative study (non-randomized)	23 patients / 27 sites	Maxilla (n=16), mandible (n=11)	Onlay bone blocks	Autologous (calvarial vs. iliac)	Titanium fixation screws and miniplates	Iliac: 13.35 mm; Calvarial: 4.22 mm	Iliac: 24.16% (pre-implant); Calvarial: 8.44%
**Gültekin et al. (2017)**	Retrospective comparative observational	39 patients / 55 sites	Atrophic maxilla	Block graft (BBG) vs ROG	BG: Autologous (iliac bone) vs ROG: particulate	BG: screws. ROG: collagen membrane (horizontal) or	BG: 8.31mm; ROG: 5.07mm	BG: 41.62% (±6.97%) volumetric resorption; ROG:

Author (Year)	Study Design	Sample (patients / sites)	Anatomical Location	Surgical Technique	Graft Material	Membrane / Device	Vertical Bone Gain (Mean)	Resorption Rate (%) / Vertical Resorption (mm)
	study (non-randomized)				autologous + xenograft (1:1)	titanium-reinformed d-PTFE membrane (vertical) with screws/tacks		15.87% (±1.99) resorption after loading
**Urban et al. (2009)**	Retrospective comparative observational study (non-randomized)	35 patients / 36 sites	Posterior maxilla (n = 8); maxilla and mandible (defects ranging from single to multiple teeth) (n = 28)	Vertical ROG	Particulate autologous bone (mandible: ramus or ching) vs autologous + xenograft (maxillary group included Bio-Oss in sinus augmentation)	Non-resorbable titanium-reinforced e-PTFE membranes, titanium tacks, and collagen membranes after removal	Autologous: 5.5 ± 2.29mm (range: 2-12mm); Autologous + xenograft: 7.4 ± 2.56mm	Mean crestal bone remodeling was 1.01 mm (±0.57) at 1 year of loading
**Barone et al. (2017)**	Retrospective comparative observational study (non-randomized)	10 patients / 20 sites	Posterior mandible	Interpositional (inlay) vs onlay	Inlay: equine cancellous bone block; Onlay: autologous block (iliac crest)	Inlay: titanium miniplates and miniscrews + autologous PRF membrane; Onlay miniscrews	Inlay (xenograft): 6.0 mm (± 0.7 mm); Onlay (autologous): 7.4mm (± 8.8mm)	Inlay XB: 35% volumetric resorption (1.7 mm); Onlay AB: 29% volumetric resorption (1.9 mm)

AB: Autologous bone; XB: Xenograft; ROG: Guided bone regeneration. BG:
