# Supplementary material for: Autologous Bone Versus Xenograft and Their Combination in Vertical Ridge Augmentation: An Analysis of Graft Resorption and Implant Survival—A Systematic Review
Source: Dent J (Basel). 2026 May 25;14(6):321. doi: 10.3390/dj14060321 (PMC13297769; doi:10.3390/dj14060321)
Supplement: Supplementary file 1 [file dentistry-14-00321-s001.zip › File S3 Implants information.pdf]

Author	Number of Implants	Follow-up Time	Marginal Bone Loss (mm)	Implant Success/Survival
**Pistilli et al., 2014**	145 implants	4 months after loading	Authors stated that peri-implant marginal bone level changes will be reported in a future publication at 12 months of loading	98.7% (AB); 82.8% (XB)
**Mazuchelli et al., 2024**	50 implants	6 months before implant placement	–	Survival rate of 98% (1 implant lost out of 50)
**Felice et al., 2009**	38 implants	1 year after loading	AB: 0.82 mm; XB (Bio-Oss): 0.59 mm	XB (Bio-Oss): 1 implant failure; overall survival approximately 97.3%
**Morad, 2013**	12 implants	4 months before implant placement	–	Not reported
**Sass et al., 2022**	57 implants	Sinus elevation: 6.2 years (range 3–12 years); Ridge augmentation: 5.9 years (range 3–11 years)	Vertical augmentation: 2.18 mm (± 0.75)	100% survival; no implant loss reported in either group
**Mertens et al., 2013**	99 implants	12 months after implant placement	No global mean MBL reported; success defined according to Albrektsson criteria (≤0.2 mm annual bone loss after the first year of function)	Calvarial: 1 implant lost (98.46% survival); Iliac crest: 100% survival
**Gültekin et al. 2017**	174 implants	30.69 months	-	ROG: 100% survival. IBG: 96.9% survival (3 implant failure)

Author	Number of Implants	Follow-up Time	Marginal Bone Loss (mm)	Implant Success/Survival
**Urban et al. 2009**	82 implants	12 and 72 months after loading	Mean crestal bone remodeling of 1.01 mm (± 0.57 mm) at 1 year of prosthetic loading	100% survival rate for the 82 implants placed. However, the cumulative success rate was 94.7%, as three implants exhibited bone remodeling greater than 2 mm, not meeting the Albrektsson criteria for success
**Barone et al. 2017**	80 implants	Radiographic evaluation: 4 months after grafting; 12 months after implant follow-up	Autologous onlay: 1.3 mm; Xenograft inlay: 0.8 mm	100% survival rate at 1 year follow-up

AB: Autologous bone; XB: Xenograft; ROG: Guided bone regeneration.
