# Supplementary material for: Autologous Bone Versus Xenograft and Their Combination in Vertical Ridge Augmentation: An Analysis of Graft Resorption and Implant Survival—A Systematic Review
Source: Dent J (Basel). 2026 May 25;14(6):321. doi: 10.3390/dj14060321 (PMC13297769; doi:10.3390/dj14060321)
Supplement: Supplementary file 1 [file dentistry-14-00321-s001.zip › File S4 Implants complications.pdf]

Study (Year)	Recipient Site Complications (dehiscence, infection, fractures)	Donor Site Complications (pain, paresthesia)	Hospital Stay (if applicable)	Total Graft Failure Rate
**Sass et al. (2022)**	None observed; no inflammatory or infectious processes during follow-up	Minimal morbidity reported for intraoral donor sites	NR (procedures under general/local anesthesia)	0%: all augmentation cases were successful in the long term
**Pistilli et al. (2014)**	15 complications in the XB group (13 dehiscences, 1 lack of integration, 1 bone sequestration)	Autologous group reported moderate pain at 10 days (10 patients) and one case of limping lasting 6 months	3.1 nights (Autologous) / 1.4 nights (Xenograft)	50% in XB group (10/20); notably 100% failure in the mandible for xenograft blocks. AB: no total failures
**Morad et al. (2013)**	No incidents; favorable soft tissue healing in all cases	No complications observed in mandibular ramus donor sites	NR	0%: all grafts allowed placement of 8–10 mm implants
**Mertens et al. (2013)**	5 dehiscences (3 calvarial, 2 iliac); two required revision due to partial loss	No complications reported in extraoral donor sites	5 days (intravenous antibiotic administration period)	0%: both groups showed 100% graft success at implant placement
**Felice et al. (2009)**	1 total failure due to fracture of the bone segment and infection (autologous group)	Iliac donor site described as technically demanding and painful	3 days (all patients)	10% (AB): one autologous graft lost; 0% (XB) for Bio-Oss blocks
**Mazuchelli et al. (2024)**	One early implant failure (2%) due to lack of patient compliance	Not reported; micrografts highlighted as reducing morbidity	NR (outpatient setting)	0%: all patients completed prosthetic rehabilitation on grafted bone

Study (Year)	Recipient Site Complications (dehiscence, infection, fractures)	Donor Site Complications (pain, paresthesia)	Hospital Stay (if applicable)	Total Graft Failure Rate
**Gültekin et al. (2017)**	1 late membrane exposure (9%) of d-PTFE in the vertical ROG group	1 patient (5%) with temporary sensory disturbance at the iliac donor site	NR (performed under general anesthesia)	0%: no cases of graft non-integration or block disintegration were reported
**Urban et al. (2009)**	1 fistula over the membrane (2.78%) at two weeks, with minimal final bone gain (2 mm)	Not reported (use of intraoral particulate graft)	NR	0% despite the complication, the site was successfully rehabilitated with implants
**Barone et al. (2017)**	4 cases of mucosal dehiscence and 1 mandibular fracture at 3 days (inlay group)	Transient paresthesia of the lower lip or chin reported in 15 patients	NR (all procedures performed under general anesthesia)	5.9% (AB): one autologous graft required removal; 0% (XB) in the inlay group

AB: Autologous bone; XB: Xenograft; NR: Not reported; GBR: Guided bone regeneration.
